# Supplementary material for: Revealing 3D structure of gluten in wheat dough by optical clearing imaging
Source: Nat Commun. 2021 Mar 17;12:1708. doi: 10.1038/s41467-021-22019-0 (PMC7969737; doi:10.1038/s41467-021-22019-0)
Supplement: Supplementary file 1 — Supplementary Information [file 41467_2021_22019_MOESM1_ESM.pdf]

Revealing 3D structure of gluten in wheat dough by optical clearing imaging

Ogawa and Matsumura

Supplementary Table 1. Variables determined by AngioTool-their application on protein networks of wheat dough and their explanations[1]. The variables used were calculated by original variables.

Original terms	Terms for protein network	Content
① Explant area		Region of interest embedded by the whole network
② Vessels area	Protein area	Area occupied by protein network
③ Vessels percentage area	Protein percentage area	Vessels area / explant area ×100
④ Total number of junctions	Protein junctions	Total number of junctions in the protein network
⑤ Junctions density	Junction density	Number of junctions / explant area
⑥ Total vessels length	Total protein length	The sum of all protein threads (distance between two junctions)
⑦ Average vessels length	Average protein length	The average length of protein threads
⑧ Total number of end points	Protein end points	Open-ended protein threads
⑨ Mean E lacunarity	Lacunarity	A measure for degree of gaps and irregularities
**Used variables**		
	Gluten percentage area	③ = ② / ① ×100
	Junction density	⑤ = ④ / ①
	Average gluten length	⑦ = ⑥ / *n*
	End points rate	⑧ / ②
	Mean E lacunarity	⑨

Supplementary Table 2. Noodle composition.

Identification code	control	10%-gluten	20%-gluten	3%-NaCl	6%-NaCl	9%-NaCl	12%-NaCl
wheat flour (g)	500	450	400	500	500	500	500
gluten powder (g)	0	50	100	0	0	0	0
water (g)	160	160	160	169	178	187	196
sodium chloride (g)	0	0	0	15	30	45	60

Supplementary Table 3. Microscope settings and imaging conditions.

			Microscope settings			Imaging conditions						
Figure			Type	Objective lens	Immersion	Excitation (nm)	Laser power (%)	Filter (nm)	XY (pixel)	XY (um/pixcel)	Z (slice)	Z (um/slice)
Fig.2	b	CLSM	Inverted	UPLSAPO 20X NA: 0.75	air	473	1.4	490-590	1,024	0.621	1	NA
	c	2PEM	Inverted	UPLSAPO10X2 NA: 0.40	air	940	37	495-540	1,024	1.242	1	NA
Fig.3	a	2PEM	Inverted	UPLSAPO10X2 NA: 0.40	air	920	30	495-540	512	2.485	1	NA
	b	2PEM	Inverted	UPLSAPO10X2 NA: 0.40	air	930	30	495-540	512	2.485	1	NA
	c	2PEM	Inverted	UPLSAPO10X2 NA: 0.40	air	940	30	495-540	512	2.485	1	NA
	d	2PEM	Inverted	UPLSAPO10X2 NA: 0.40	air	950	30	495-540	512	2.485	1	NA
	e	2PEM	Inverted	UPLSAPO10X2 NA: 0.40	air	960	30	495-540	512	2.485	1	NA
	g	2PEM	Inverted	UPLSAPO10X2 NA: 0.40	air	820	30	647/57	512	2.485	1	NA
	h	2PEM	Inverted	UPLSAPO10X2 NA: 0.40	air	830	30	647/57	512	2.485	1	NA
	i	2PEM	Inverted	UPLSAPO10X2 NA: 0.40	air	840	30	647/57	512	2.485	1	NA
	j	2PEM	Inverted	UPLSAPO10X2 NA: 0.40	air	850	30	647/57	512	2.485	1	NA
	k	2PEM	Inverted	UPLSAPO10X2 NA: 0.40	air	860	30	647/57	512	2.485	1	NA
	m	2PEM	Upright	XLSLPlan N 25X NA: 1.00	SoROCS	940	9	520-560	1,600	0.212	1	NA
	m	2PEM	Upright	XLSLPlan N 25X NA: 1.00	SoROCS	840	9	647/57	1,600	0.212	1	NA
Fig.4	b, c left	2PEM	Upright	XLPLN 25X W NA: 1.05	Water	940	2.0–2.5	495-540	640	0.795	71	2.5
	b, c right	2PEM	Upright	XLSLPlan N 25X NA: 1.00	SoROCS	940	10–25	495-540	640	0.795	882	2.5
	e	2PEM	Upright	XLSLPlan N 25X NA: 1.00	SoROCS	940	11	495-540	640	0.795	1	NA
	f	2PEM	Upright	XLSLPlan N 25X NA: 1.00	SoROCS	940	20	495-540	640	0.795	1	NA
	g	2PEM	Upright	XLSLPlan N 25X NA: 1.00	SoROCS	940	55	495-540	640	0.795	1	NA
	h	2PEM	Upright	XLSLPlan N 25X NA: 1.00	SoROCS	940	66	495-540	640	0.795	1	NA
Fig.5	a	2PEM	Upright	XLSLPlan N 25X NA: 1.00	SoROCS	940	27	495-540	640	0.795	340	1.5
	b	2PEM	Upright	XLSLPlan N 25X NA: 1.00	SoROCS	940	24–31	495-540	640	0.795	340	1.5
	c	2PEM	Upright	XLSLPlan N 25X NA: 1.00	SoROCS	940	28–49	495-540	640	0.795	340	1.5
	j	2PEM	Upright	XLSLPlan N 25X NA: 1.00	SoROCS	940	30–38	495-540	640	0.795	340	1.5
	k	2PEM	Upright	XLSLPlan N 25X NA: 1.00	SoROCS	940	34–40	495-540	640	0.795	340	1.5
	l	2PEM	Upright	XLSLPlan N 25X NA: 1.00	SoROCS	940	30–36	495-540	640	0.795	340	1.5
	m	2PEM	Upright	XLSLPlan N 25X NA: 1.00	SoROCS	940	28–33	495-540	640	0.795	340	1.5
Fig.7	b	CLSM	Inverted	UPLSAPO 20X NA: 0.75	air	473	5–50	490-590	640	0.994	311	2
	c	CLSM	Inverted	UPLSAPO 20X NA: 0.75	air	473	23	490-590	640	0.994	1	NA
	d	CLSM	Inverted	UPLSAPO 20X NA: 0.75	air	473	59	490-590	640	0.994	1	NA
Sup. Fig.3	a	CLSM	Inverted	UPLSAPO 20X NA: 0.75	air	473	1	490-540	512	1.242	1	NA
	b	CLSM	Inverted	UPLSAPO 20X NA: 0.75	air	559	18	575-675	512	1.242	1	NA

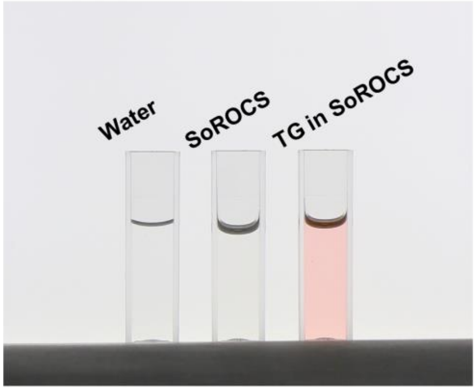

Supplementary Fig. 1. Appearance of SoROCS. SoROCS is nearly colourless and transparent and appears as a slight pink colour when mixed with 0.0005% TG. Since TG is pale in colour despite its strong fluorescence, light is less likely to be scattered when observed under a microscope.

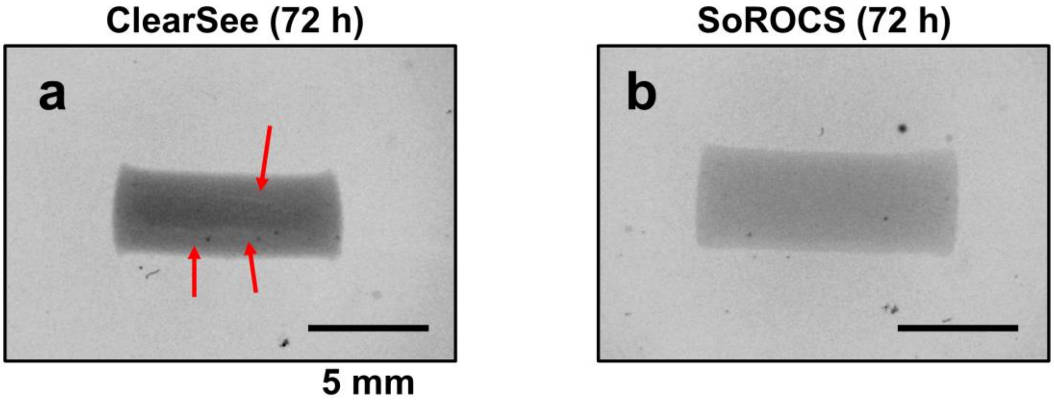

Supplementary Fig. 2. Effect of clearing reagents on sample fragility. Transparent image of noodles cleared with ClearSee (**a**) and SoROCS (**b**). Note, the images of **a** and **b** are the same as in Fig. 1g and h, respectively, however, the image processing was performed in the same manner for both images to improve visibility. Scale bars indicate 5 mm. Red arrows indicate visible crevices.

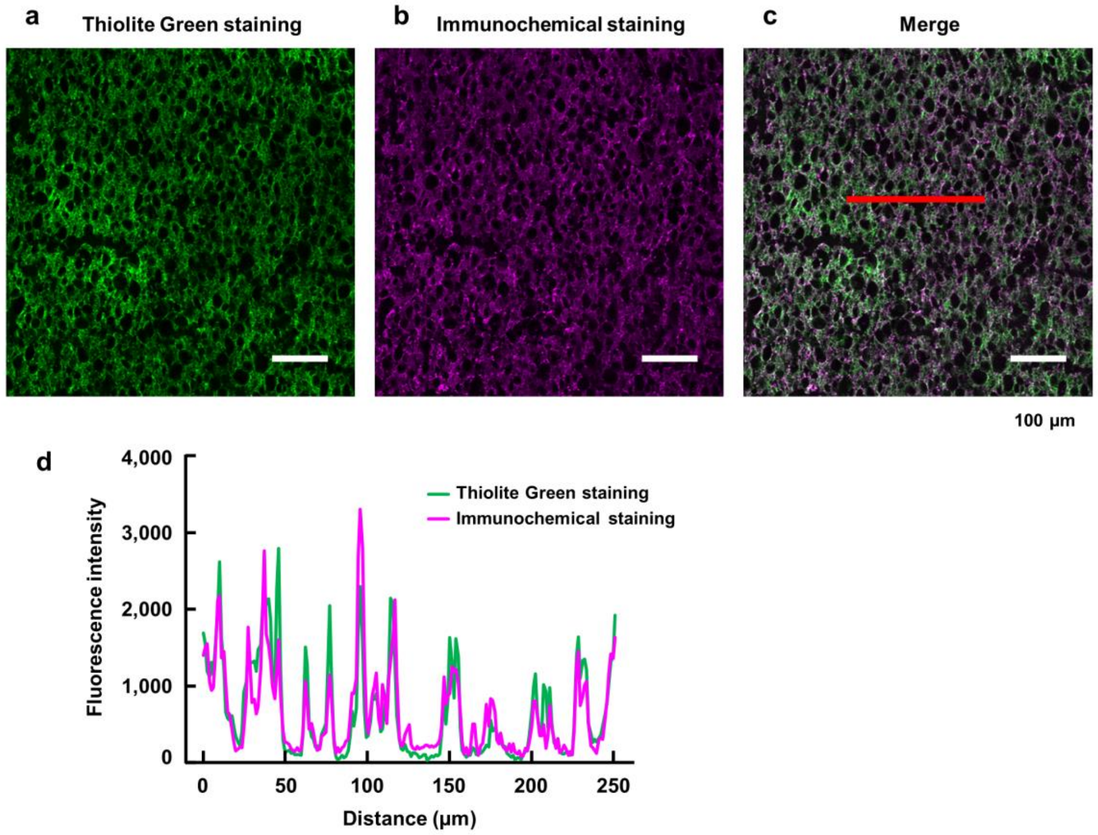

Supplementary Fig. 3. Specificity of gluten staining with TG. Non-specific staining with TG image (**a**), gluten-specific immunochemical staining image (**b**); merged image of **a** and **b** (**c**). **d** Plot profile of fluorescence intensity for TG staining (green) and immunochemical staining (magenta) along the red line shown in **c**. The plot profile analysis on an image for gluten which was co-stained with fluorescently labelled antibody and TG showed that the tops of the peaks overlapped with each other, indicating that TG can depict gluten structures in noodles. This experiment was repeated three times using independently prepared noodle samples with similar results. Source data underlying Supplementary Figure 3d are provided as a Source Data file.

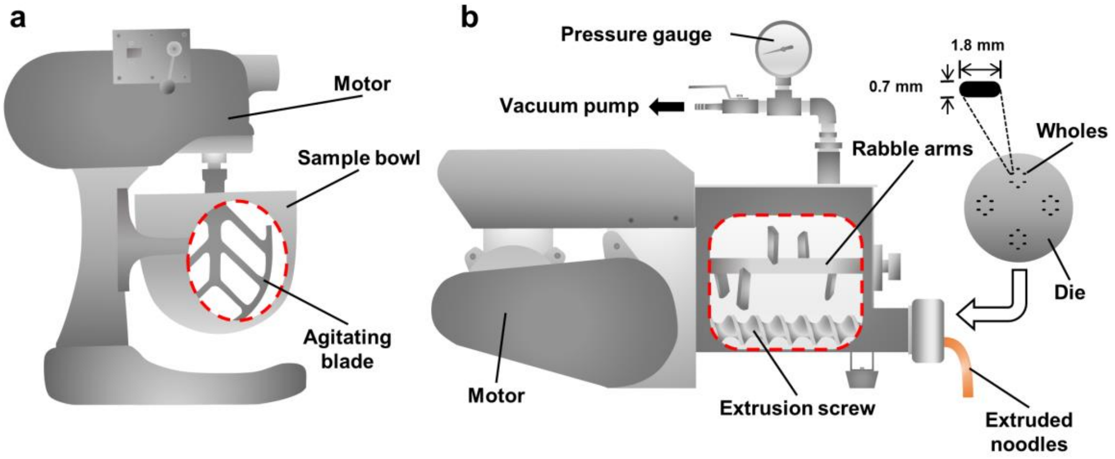

Supplementary Fig. 4. External view of the noodle machine. a Hobart mixer, which kneaded the mixture of wheat flour and excess gluten powder with Milli-Q water or sodium chloride solutions in the sample bowl to produce a wheat dough. The wheat dough was agitated with a blade for 20 min to uniformly distribute the water. **b** A pasta-making machine equipped with a vacuum pump (not depicted in the figure) extruded noodles with an extrusion screw through holes in a Teflon die under reduced pressure. The portions surrounded by the red dotted line depict the inside of each device (**a**, **b**).

Supplementary Reference

1. Bernklau, I., Lucas, L., Jekle, M. & Becker, T. Protein network analysis-A new approach for quantifying wheat dough microstructure. *Food Res. Int.* **89**, 812−819 (2016).
