# Supplementary material for: Revealing 3D structure of gluten in wheat dough by optical clearing imaging
Source: Nat Commun. 2021 Mar 17;12:1708. doi: 10.1038/s41467-021-22019-0 (PMC7969737; doi:10.1038/s41467-021-22019-0)
Supplement: Supplementary file 3 — Reporting Summary [file 41467_2021_22019_MOESM3_ESM.pdf]

nature research

Corresponding author(s): Takenobu Ogawa and Yasuki Matsumura

Last updated by author(s): Mar 5, 2021

Reporting Summary

Nature Research wishes to improve the reproducibility of the work that we publish. This form provides structure for consistency and transparency in reporting. For further information on Nature Research policies, see our Editorial Policies and the Editorial Policy Checklist.

Statistics

For all statistical analyses, confirm that the following items are present in the figure legend, table legend, main text, or Methods section.

n/a	Confirmed	
☐	☒	The exact sample size (*n*) for each experimental group/condition, given as a discrete number and unit of measurement
☐	☒	A statement on whether measurements were taken from distinct samples or whether the same sample was measured repeatedly
☐	☒	The statistical test(s) used AND whether they are one- or two-sided<br>*Only common tests should be described solely by name; describe more complex techniques in the Methods section.*
☒	☐	A description of all covariates tested
☒	☐	A description of any assumptions or corrections, such as tests of normality and adjustment for multiple comparisons
☐	☒	A full description of the statistical parameters including central tendency (e.g. means) or other basic estimates (e.g. regression coefficient) AND variation (e.g. standard deviation) or associated estimates of uncertainty (e.g. confidence intervals)
☐	☒	For null hypothesis testing, the test statistic (e.g. *F*, *t*, *r*) with confidence intervals, effect sizes, degrees of freedom and *P* value noted<br>*Give P values as exact values whenever suitable.*
☒	☐	For Bayesian analysis, information on the choice of priors and Markov chain Monte Carlo settings
☒	☐	For hierarchical and complex designs, identification of the appropriate level for tests and full reporting of outcomes
☒	☐	Estimates of effect sizes (e.g. Cohen's *d*, Pearson's *r*), indicating how they were calculated

Our web collection on statistics for biologists contains articles on many of the points above.

Software and code

Policy information about availability of computer code

Data collection	Imaging for opacity: ImageQuant LAS 4000 with control software Ver. 1.2 (GE Healthcare)<br>Imaging for morphology: EOS 70D and Digital Photo Professional Ver. 4.3.31.0 for collection and Ver. 4.12.60.0 for data output (Canon)<br>Microscopy: FV1200MPE-BX61WI, FV1000MPE-IX83, and FV1000D-IX81 with Fluoview FV10-ASW Ver. 4.2c for FV1200MPE-BX61WI and FV1000MPE-IX83; Ver. 3.0a for FV1000D-IX81 (Olympus), and with FV31S-SW Ver. 2.3.1.163 for data output (Olympus)<br>Compression test: RE2-33005B Rheoner II (Yamaden)
Data analysis	2D image quantification: Fiji (ImageJ Ver. 1.52i)<br>3D image quantification: Imaris x64 Ver. 8.3.1 (Bitplane)<br>3D image construction and reslice: FluoRender Ver. 2.23.0 and Imaris x64 Ver. 8.3.1 (Bitplane)<br>Calculation of numerical data and statistical analysis: Microsoft 365 Excel<br>Point-spread-function analysis: OriginPro 8.1J SR3 Ver. 8.1.34.90 (OriginLab)<br>Protein network analysis: AngioTool64 Ver. 0.6a

For manuscripts utilizing custom algorithms or software that are central to the research but not yet described in published literature, software must be made available to editors and reviewers. We strongly encourage code deposition in a community repository (e.g. GitHub). See the Nature Research guidelines for submitting code & software for further information.

April 2020

Data

Policy information about availability of data

All manuscripts must include a data availability statement. This statement should provide the following information, where applicable:

- Accession codes, unique identifiers, or web links for publicly available datasets
- A list of figures that have associated raw data
- A description of any restrictions on data availability

Data supporting the findings of this work are available within the paper and its Supplementary Information files. The image data sets are available at http://doi.org/10.5281/zenodo.4540950. The source data underlying Figures 1i, m-o, 2a, 2f-j, 3f, l, o, 4d, 5n, 6d, and 7e as well as Supplementary Figures 3d are provided as a Source Data file. Source data file are provided with this paper.

Field-specific reporting

Please select the one below that is the best fit for your research. If you are not sure, read the appropriate sections before making your selection.

☒ Life sciences ☐ Behavioural & social sciences ☐ Ecological, evolutionary & environmental sciences

For a reference copy of the document with all sections, see nature.com/documents/nr-reporting-summary-flat.pdf

Life sciences study design

All studies must disclose on these points even when the disclosure is negative.

Sample size	No statistical method was used to predetermine sample size. The sample sizes for opacity and linear expansion experiments were determined based on previous publications (e.g. DOI: 10.1038/nn.3447). The sample sizes for other experiments were determined from preliminary experiments and was based on the reproducibility between independent experiments. The number of independent experiments and replicates is indicated in the figure legends and the main manuscript.
Data exclusions	No data was excluded.
Replication	Similar experimental results have been obtained over multiple measurements with reported sample sizes in the figure legends and the main manuscript. All replication attempts were successful.
Randomization	As our data do not include clinical trials or related experiments, randomization was not applied. For experiments involving noodle clearing, all different conditions were randomly placed at the same time and all experimental procedures were strictly similar between the different conditions.
Blinding	No blinding method was possible as only one experimenter was performing the analyses.

Reporting for specific materials, systems and methods

We require information from authors about some types of materials, experimental systems and methods used in many studies. Here, indicate whether each material, system or method listed is relevant to your study. If you are not sure if a list item applies to your research, read the appropriate section before selecting a response.

Materials & experimental systems

n/a	Involved in the study	
☐	☒	Antibodies
☒	☐	Eukaryotic cell lines
☒	☐	Palaeontology and archaeology
☒	☐	Animals and other organisms
☒	☐	Human research participants
☒	☐	Clinical data
☒	☐	Dual use research of concern

Methods

n/a	Involved in the study	
☒	☐	ChIP-seq
☒	☐	Flow cytometry
☒	☐	MRI-based neuroimaging

Antibodies

Antibodies used	Chicken polyclonal anti-wheat gluten (for ELISA, Western blot), Agrisera, cat# AS09 571, Lot 2008<br>Goat anti-chicken IgY (H+L) secondary antibody with Alexa Fluor 594, Invitrogen, cat# A11042, Lot 2092259
Validation	All antibodies were used under manufacturer's recommendations. And the dilution ratios are mentioned in methods.<br><br>Wheat gluten (https://www.agrisera.com/en/artiklar/wheat-gluten.html)

Goat anti-chicken IgY (H+L) secondary antibody with Alexa Fluor 594 (https://www.thermofisher.com/antibody/product/Goat-anti-Chicken-IgY-H-L-Secondary-Antibody-Polyclonal/A-11042)
